# Deep Generative Modeling: From Probabilistic Framework to Generative AI

**DOI:** 10.3390/e27030238

**Published:** 2025-02-26

**Authors:** Jakub M. Tomczak

**Affiliations:** Chan Zuckerberg Initiative, Redwood City, CA 94063, USA; jmk.tomczak@gmail.com

## 1. Why *Deep Generative Modeling*?

Large Language Models (LLMs) have unlocked a new frontier in AI applications, significantly advancing the field of generative modeling. Beyond assisting with tasks such as preparing a presentation plan, LLMs have fundamentally reshaped human–computer interactions by enabling seamless communication between machines and agents through natural language. This paradigm shift has not only driven a technological leap but has also transformed how we conceptualize AI and its role in solving complex problems.

The recent advancements in Generative AI (GenAI), such as Vision-Language Models (VLMs), exemplified by systems like GPT-4V (GPT-4 with Vision) [1] and Google’s Gemini [2], have brought multimodal learning back into the spotlight. These developments underscore the versatility of LLMs, which now form the core of multimodal systems capable of processing and generating diverse data types—including text, images, audio, and video. The fusion of modalities within these systems has set the stage for a new generation of AI termed Generative AI Systems (GenAISys) [3].

GenAISys are holistic systems where natural language serves as the universal medium for communication, replacing predefined formal protocols. These systems integrate modality encoders as I/O interfaces to handle various data sources. Furthermore, they incorporate databases, knowledge graphs, and external specialized tools—such as calculators, navigation apps, or molecular drawing utilities—that communicate with the system through dedicated modules for information retrieval and storage. While GenAI models remain central to GenAISys, they function as components within a broader, more interconnected framework rather than standalone entities.

For instance, a tool-augmented Seq2Seq model proposed by [4] uses a fine-tuned T5 LLM as a backbone to interface with external tools. Similarly, ref. [5] illustrates a GenAISys where an LLM collaborates with tools and databases to answer chemistry-related questions and generate molecular diagrams. These examples highlight the emergent capabilities of GenAISys in addressing domain-specific challenges while leveraging the general-purpose strengths of LLMs.

However, the increasing complexity of these systems necessitates a shift in perspective. Instead of viewing GenAISys as collections of separate modules, we must analyze them as cohesive systems. This shift invites us to examine their compositionality [6,7,8,9], i.e., how individual components interact to produce coherent and reliable outcomes. Insights from compositionality studies can guide the design of robust and verifiable systems, drawing parallels to disciplines like control theory and systems engineering [10].

In addition to compositionality, grounding generative systems in robust theoretical foundations is essential. This involves leveraging modeling principles that capture the generative processes underlying observed phenomena. Such processes define a joint distribution over random variables and their stochastic interactions, specifying how events occur and in what sequence. To create flexible, efficient, and effective modeling frameworks, these distributions can be parameterized using deep neural networks capable of processing raw data.

By modeling stochastic dependencies through probability theory, we ensure a rigorous and principled approach, minimizing potential reasoning errors. Probability theory also offers a unified framework in which the likelihood function is central to quantifying uncertainty and defining objective functions. This approach, known as deep generative modeling [11], integrates these concepts into a cohesive framework, providing a powerful foundation for the development of Generative AI Systems.

Moreover, addressing practical concerns such as scalability, interpretability, and ethical considerations is also crucial. Applying these principles to GenAISys can ensure they meet the high expectations placed upon them while avoiding potential pitfalls.

Looking ahead, the exploration of GenAISys offers an opportunity to refine our understanding of AI systems as interconnected ecosystems. By investigating their compositional, functional, and ethical dimensions, we can design more effective, reliable, and human-centric AI solutions, pushing the boundaries of what is achievable in this exciting era of Generative AI.

## 2. Formulating and Applying *Deep Generative Modeling*

There are multiple classes of (deep) generative models, namely, mixture models, Probabilistic Circuits, Autoregressive Models (and their special example, Large Language Models), Flow-based Models, Latent Variable Models, GANs, Hybrid (or Joint) Models, Score-based Generative Models, Diffusion-based Models, and Energy-based Models. All these classes utilize and rely on various probabilistic principles, e.g., normalizing flows are built upon the change in variable formula. However, their formulation depends on deep neural network architectures. For instance, Large Language Models are Autoregressive Models parameterized by transformers or recurrent neural networks, while normalizing flows require invertible deep neural networks.

The objective of this Special Issue is to provide the latest advances in *Deep Generative Modeling* both in theory and applications. In the following articles, various deep generative models are presented (e.g., diffusion models, Variational Auto-Encoders, normalizing flows, etc.) and applied to applications like image and video generation, tabular data processing, and microRNA generation. Additionally, some studies consider the theoretical aspects of these models, such as the number of steps in diffusion-based models, a connection between diffusion-based models and associative memory, or the new Generative Bayesian Framework.

The future seems significantly dependent on *Deep Generative Modeling* and Generative AI Systems that comprise the inevitable next steps in the evolution of AI. They will assist in many jobs, ranging from office jobs, healthcare, and education to industries like manufacturing. Although there are many other aspects like embodied AI [12] or cyber–physical systems (with humans) [9], GenAISys will still be necessary for formulating an artificial “brain” and/or GenAI-based agents and apps.

## 3. About This Special Issue

This Special Issue “Deep Generative Modeling: Theory and Applications” presents a collection of research papers that explore advancements in deep generative models and their diverse applications. Notable contributions include the development of Generative Bayesian Computation methods for efficient maximum-expected utility calculations, the use of Variational Auto-Encoders for generating descriptions for precursor microRNA aiding in gene regulation studies, and the introduction of Dimma, a semi-supervised approach for enhancing low-light images with natural colors. This Special Issue also features NodeFlow, a framework combining Neural Oblivious Decision Ensembles and Conditional Continuous Normalizing Flows for probabilistic regression on tabular data, and studies on diffusion-based causal representation learning, highlighting the theoretical and practical advancements in the field of deep generative modeling. Moreover, this collection features other notable papers advancing the field of deep generative models. The study on diffusion models as associative memory networks explores the connection between generative diffusion models and associative memory networks, proposing a framework that unifies creative generation and memory recall processes. Other significant contributions include studies on multi-modal latent diffusion, semi-supervised variational autoencoders for out-of-distribution generation, diffusion probabilistic modeling for video generation, learning energy-based models in high-dimensional spaces with multiscale denoising-score matching, and investigations into optimal diffusion times in score-based generative models. These works collectively enhance our understanding and application of deep generative modeling techniques.
